# PM_2.5_ exposure and DLEC1 promoter methylation in Taiwan Biobank participants

**DOI:** 10.1186/s12199-020-00909-x

**Published:** 2020-11-05

**Authors:** Ying-Hsiang Chou, Disline Manli Tantoh, Ming-Chi Wu, Yeu-Sheng Tyan, Pei-Hsin Chen, Oswald Ndi Nfor, Shu-Yi Hsu, Chao-Yu Shen, Chien-Ning Huang, Yung-Po Liaw

**Affiliations:** 1 Institute of Medicine, Chung Shan Medical University, Taichung City, 40201 Taiwan; 2 School of Medical Imaging and Radiological Sciences, Chung Shan Medical University, Taichung City, 40201 Taiwan; 3 Department of Radiation Oncology, Chung Shan Medical University Hospital, Taichung, 40201 Taiwan; 4 Department of Medical Imaging, Chung Shan Medical University Hospital, No. 110, Sec. 1 Jianguo N. Rd, Taichung City, 40201 Taiwan; 5 Department of Public Health and Institute of Public Health, Chung Shan Medical University, No. 110, Sec. 1 Jianguo N. Rd, Taichung City, 40201 Taiwan; 6 School of Medicine, Chung Shan Medical University, Taichung City, 40201 Taiwan; 7 School of Medical Informatics, Chung Shan Medical University, Taichung City, 40201 Taiwan; 8 Department of Internal Medicine, Chung-Shan Medical University Hospital, Taichung City, 40201 Taiwan; 9 Medical Imaging and Big Data Center, Chung Shan Medical University Hospital, Taichung City, 40201 Taiwan

**Keywords:** PM_2.5_, DLEC1, Methylation, Exercise, Taiwan Biobank

## Abstract

**Background:**

Particulate matter (PM) < 2.5 μm (PM_2.5_) or fine PM is a serious public health concern. It affects DNA methylation and heightens carcinogenesis. Deleted in lung and esophageal cancer 1 (DLEC1) is a tumor suppressor gene. However, aberrant methylation of the gene is associated with several cancers. We evaluated the association between PM_2.5_ and DLEC1 promoter methylation in Taiwanese adults based on regular outdoor exercise.

**Methods:**

We obtained DNA methylation and exercise data of 496 participants (aged between 30 and 70 years) from the Taiwan Biobank (TWB) database. We also extracted PM_2.5_ data from the Air Quality Monitoring Database (AQMD) and estimated participants’ exposure using residential addresses.

**Results:**

DLEC1 methylation and PM_2.5_ were positively associated: beta coefficient (*β*) = 0.114 × 10^−3^; *p* value = 0.046. The test for interaction between exercise and PM_2.5_ on DLEC1 methylation was significant (*p* value = 0.036). After stratification by exercise habits, PM_2.5_ and DLEC1 methylation remained significantly associated only among those who exercised regularly (*β* = 0.237 × 10^−3^; *p* value = 0.007). PM_2.5_ quartile-stratified analyses revealed an inverse association between regular exercise and DLEC1 methylation at PM_2.5_ < 27.37 μg/m^3^ (*β* = − 5.280 × 10^−3^; *p* value = 0.009). After combining exercise habits and PM_2.5_ quartiles, one stratum (i.e., regular exercise and PM_2.5_ < 27.37 μg/m^3^) was inversely associated with DLEC1 methylation (*β* = -5.160 × 10^−3^, *p* value = 0.007).

**Conclusions:**

We found significant positive associations between PM_2.5_ and DLEC1 promoter methylation. Regular exercise at PM_2.5_ < 27.37 μg/m^3^ seemingly regulated DLEC1 promoter methylation.

## Introduction

PM_2.5_ induces the generation of reactive oxygen species (ROS) which have detrimental effects [1], like immune response stress, inflammatory injury, DNA damage, and oxidative stress that enhance cancer formation [1–3]. PM_2.5_ is a critical public health issue that accounts for most air pollution-related global deaths [1, 4]. It accounted for approximately 3.5 and 4.2 million global deaths in 1990 and 2015, respectively. Moreover, it was the fifth top cause of global mortality in 2015 [5]. While PM_2.5_ could enhance inflammation and oncogenesis [3], exercise, on the other hand, could curb inflammation and related complications [6–9]. All mechanisms by which PM_2.5_ and exercise affect tumorigenesis are not yet delineated.

DNA methylation is an epigenetic change that integrates the interactions between genes and the environment [10]. Abnormal patterns of this epigenetic marker are promising diagnostic and prognostic tumor markers because they are frequently detected even at the earliest stages of tumor formation [11, 12]. DNA methylation is one of the epigenetic mechanisms underlying the incidence of air pollution-induced allergic diseases [10] and human tumors [13, 14]. PM_2.5_-induced DNA methylation alterations aggravate the risk of cancer by repressing tumor suppressor genes and activating oncogenes [15]. DNA methylation is believed to be an epigenetic indicator of exercise intervention [16, 17]. For instance, in a study where exercise-induced immunologic benefits attenuated the detrimental effects of air pollution on the lungs, DNA methylation was a marker of such benefits [18].

Deleted in lung and esophageal cancer 1 (DLEC1) is an important element in head and neck tumorigenesis [13, 19–22]. It is a bona fide tumor suppressor gene located on chromosome 3p22.3 [13, 19–24]. Its tumor-suppressing potentials were initially identified in lung, renal, and esophageal carcinomas [21]. Abnormalities in DLEC1 methylation are potential diagnostic and prognostic epigenetic tumor markers [25]. For instance, DLEC1 promoter methylation is a prognostic biomarker for gastric, lung, advanced ovarian, and endometrial cancer [11, 26–31]. It is also a diagnostic biomarker for prostate, breast, ovarian, colorectal, gastric, nasopharyngeal, and lung cancer [11, 25, 30, 32–39].

Findings on the relationship between air pollution and DNA methylation warrant critical replication and validation [40]. Exposure to smoky coals was associated with higher DLEC1 methylation in plasma and tissue samples from Chinese lung cancer patients [32], implying that air pollution-induced DLEC1 methylation could influence cancer etiology. Even though both DLEC1 methylation and PM_2.5_ play prominent roles in carcinogenesis, their relationship has received limited research attention. Moreover, studies on DLEC1 methylation among Taiwanese are lacking. Furthermore, the combined effects of exercise and PM_2.5_ exposure on health, alongside the underlying mechanisms require more investigations [8, 16, 41]. We evaluated the association between PM_2.5_ and DLEC1 promoter methylation in relation to exercise among TWB participants.

## Methods

### Data sources and study population

We obtained data from two sources, namely the TWB database and AQMD. Recruitment of participants into the TWB project is restricted to Taiwanese aged between 30 and 70 years who have never been diagnosed with cancer. The TWB has 29 recruitment centers throughout Taiwan [42]. The TWB database (2008 to 2015) has data on methylation, exercise, sex, age, cigarette smoking, weight, height, secondhand smoke exposure, and alcohol/betel nut intake. Five DLEC1 promoter CpG sites (cg04833533, cg16150706, cg11542528, cg20684180, and cg23881725) were available in the TWB database. It is worth stating that this dataset does not have air pollution data. So, we obtained annual averages (2006–2011) of daily concentrations of PM_2.5_ (μg/m^3^), SO_2_ (ppb), CO (ppm), O_3_ (ppb), and NOx (ppb) from the AQMD. The Environmental Protection Administration (EPA) in Taiwan monitors air quality through the Air Quality Monitoring Network (AQMN) [43, 44]. To date, the EPA has set up about 77 fully automated air quality monitoring stations nationwide for daily monitoring of air pollution [44]. We included 496 individuals with complete data in the final analyses.

### Main exposure and outcome

The main exposure was PM_2.5_ while the outcome was DLEC1 promoter methylation. We used the residential addresses of participants and estimated their PM_2.5_ exposure. Health GeneTech Corp. performed all the DNA methylation experiments. In brief, a trained and qualified researcher with a medical background collected about 9 ml of venous blood from each participant into sodium citrate tubes. The blood samples were kept at 4 °C and transported to the laboratory for further experiments. DNA isolation and purification was done using an automated chemical extraction instrument called Chemagic™ Prime™. Purified DNA samples were treated with sodium bisulfite using EZ DNA Methylation Kit (Zymo Research, CA, USA). DNA methylation was assessed with the Infinium® MethylationEPIC BeadChipEPIC array (Illumina Inc.) and presented as beta values, which range from 0 to 1 [45, 46]. Lower and higher beta values indicate lower and higher methylation levels, respectively. The Infinium® MethylationEPIC BeadChipEPIC array targets more than 850,000 CpG sites across the genome. Quality control measures for methylation data were untaken as previously stated [47, 48]. Ethical approval for this study was given by the Institutional Review Board of Chung Shan Medical University Hospital (CS2-20007).

### Covariates

We used participants’ residential addresses and estimated the annual average exposure levels of SO_2_ (ppb), CO (ppm), O_3_ (ppb), and NOx (ppb). Self-filled TWB questionnaires contained data on exercise, sex, age, cigarette smoking, betel nut chewing, alcohol intake, and secondhand smoke exposure. Detailed descriptions of these variables and BMI are found somewhere else [47, 48]. Summarily, we defined outdoor exercise as engaging in any outdoor activities (e.g., Chinese martial arts, “Wai-Tan-Kung,” “Neidan-Kung,” “Falun Dafa,” “Taijiquan,” “Xiang Kong,” “Yuan Chin Dance,” “Qigong,” strolling, jogging, hiking, rope jumping, arm swing, soccer, golf, croquet, tennis, basketball, other ball games, biking, mountain climbing, and hula hoop) lasting over 30 min, at least three times per week.

### Statistical analyses

We stratified the basic characteristics of the participants into regular and no regular exercise. DLEC1 promoter methylation (mean ± standard error) was the average of the beta values of the 5 DLEC1 promoter CpG sites. We evaluated the differences in continuous variables (DLEC1 methylation levels, PM_2.5_, SO_2_, CO, O_3_, NOx, and age) between the two exercise groups with the *T* test and the differences in the categorical variables (sex, cigarette smoking, alcohol/betel nut intake, BMI, and exposure to secondhand smoke) between the two groups with the chi-square test. Moreover, we determined the association between PM_2.5_ and DLEC1 promoter methylation by employing multivariate linear regression analysis and adjusted for covariates (consisting of exercise, SO_2_, CO, O_3_, NOx, sex, age, cigarette smoking, BMI, secondhand smoke exposure, and alcohol/betel nut intake). We also used multivariate linear regression and determined the interaction between PM_2.5_ and exercise on DLEC1 promoter methylation. We adjusted for cell-type composition in whole blood using the Reference-Free Adjustment for Cell-Type composition (ReFACTor) method [49]. For all analyses, a *p* value < 0.05 was considered statistically significant. All statistical analyses were executed with the SAS software; version 9.4 (SAS Institute Inc., Cary, NC, USA).

## Results

We included a total of 496 participants (i.e., 211 with regular and 285 with no regular exercise habits). DLEC1 promoter methylation, age, cigarette smoking, BMI, and secondhand smoke exposure were significantly different (*p* value < 0.050) between the exercise groups (Table 1).

**Table 1:** Demographic characteristics of participants stratified by regular and no regular exercise

Variables	No regular exercise *n* = 285	Regular exercise *n* = 211	*p* value
Mean DLEC1 promoter methylation (beta value)	0.198 ± 0.001	0.193 ± 0.001	< 0.001
PM_2.5_ (μg/m^3^)	31.551 ± 0.455	32.756 ± 0.550	0.090
SO_2_ (ppb)	4.137 ± 0.092	4.092 ± 0.099	0.741
CO (ppm)	0.560 ± 0.013	0.546 ± 0.013	0.450
O_3_ (ppb)	27.860 ± 0.192	27.989 ± 0.221	0.660
NO_X_ (ppb)	26.411 ± 0.746	25.581 ± 0.794	0.453
Sex			0.075
Women	150 (52.63)	94 (44.55)	
Men	135 (47.37)	117 (55.45)	
Age (years)	45.832 ± 0.635	55.185 ± 0.660	< 0.001
Cigarette smoking			0.011
Never	222 (77.89)	152 (72.04)	
Former	27 (9.47)	39 (18.48)	
Current	36 (12.63)	20 (9.48)	
Alcohol drinking			0.633
Never	261 (91.58)	188 (89.10)	
Former	8 (2.81)	7 (3.32)	
Current	16 (5.61)	16 (6.50)	
Betel nut chewing			0.516
No	266 (93.33)	202 (95.73)	
Former	11 (3.86)	5 (2.37)	
Current	8 (2.81)	4 (1.90)	
BMI (kg/m^2^)			0.040
Normal (< 18.5)	130 (45.61)	94 (44.55)	
Underweight (18.5 ≤ BMI < 24)	17 (5.96)	3 (1.42)	
Overweight (24 ≤ BMI < 27)	76 (26.67)	71 (33.65)	
Obesity (BMI ≥ 27)	62 (21.75)	43 (20.38)	
Secondhand smoke exposure			0.009
No	247 (86.67)	198 (93.84)	
Yes	38 (13.33)	13 (6.16)	

Standard error (SE) represents continuous variables and percentage (%) represents categorical variables

Air pollutant concentrations are annual averages (2006–2011) of daily concentrations. *DLEC1* Deleted in lung and esophageal cancer 1, *PM* Particulate matter with diameter less than 2.5 microns, *SO*_*2*_ Sulfur dioxide, *CO* Carbon monoxide, *O*_*3*_ Ozone, *NOx* Nitrogen oxides, *BMI* Body mass index

PM_2.5_ was significantly associated with hypermethylation or higher levels (*β* = 0.114 × 10^−3^; *p* value = 0.046) while age was significantly associated with hypomethylation or lower levels of DLEC1 promoter methylation: *β* = − 0.206 × 10^−3^; *p* value = < 0.001 (Table 2).

**Table 2:** Multiple linear regression showing the association of PM_2.5_ and outdoor exercise with DLEC1 promoter methylation in participants

Variables	*β*(× 10^−3^)	*p* value
PM_2.5_	0.114	0.046
Regular outdoor exercise (reference: No)		
Yes	− 1.080	0.246
SO_2_	0.321	0.323
CO	− 9.120	0.199
O_3_	0.030	0.889
NO_X_	0.119	0.330
Sex (reference: Women)		
Men	0.513	0.589
Age	− 0.206	< 0.001
Cigarette smoking (reference: Never)		
Former	1.560	0.250
Current	2.590	0.088
Alcohol drinking (reference: Never)		
Former	− 3.400	0.176
Current	1.790	0.309
Betel nut chewing (reference: Never)		
Former	− 1.560	0.524
Current	− 3.990	0.175
BMI (reference: Normal)		
Underweight	− 0.725	0.739
Overweight	− 0.092	0.926
Obesity	1.760	0.113
Secondhand smoke exposure (reference: No)		
Yes	− 0.258	0.855

*DLEC1* Deleted in lung and esophageal cancer 1, *PM* Particulate matter with diameter less than 2.5 microns, *SO*_*2*_ Sulfur dioxide, *CO* Carbon monoxide, *O*_*3*_ Ozone, *NOx* Nitrogen oxides, *BMI* Body mass index

Exercise and PM_2.5_ had a significant interaction on DLEC1 methylation (*p* value = 0.036). When we stratified participants by regular exercise habits, PM_2.5_ and DLEC1 methylation remained positively associated. However, this association was significant only in the regular exercise group (*β* = 0.237 × 10^−3^, *p* value = 0.007). The inverse association between age and DLEC1 methylation remained significant in both exercise groups. The *β* coefficient was − 0.212 × 10^−3^ (*p* value < 0.001) for regular exercise and − 0.144 × 10^−3^ (*p* value = 0.042) for no regular exercise (Table 3). There was a significant association between obesity and DLEC1 methylation in those who exercised regularly: *β* = 3.870 × 10^−3^, *p* value = 0.029.

**Table 3:** Multiple linear regression showing the association between PM_2.5_ and DLEC1 promoter methylation based on regular outdoor exercise habits

Variables	No regular outdoor exercise		Regular outdoor exercise	
	*β*(× 10^−3^)	*p* value	*β*(× 10^−3^)	*p* value
PM_2.5_	0.012	0.880	0.237	0.007
SO_2_	0.251	0.535	0.505	0.357
CO	1.250	0.890	− 25.170	0.033
O_3_	0.345	0.224	− 0.607	0.078
NO_X_	0.045	0.771	0.203	0.314
Sex (reference: Women)				
Men	1.220	0.322	− 0.897	0.567
Age	− 0.212	< 0.001	− 0.144	0.042
Cigarette smoking (reference: Never)				
Former	0.613	0.761	2.210	0.256
Current	3.050	0.104	0.412	0.880
Alcohol drinking (reference: Never)				
Former	− 4.540	0.186	0.435	0.910
Current	1.580	0.527	2.670	0.295
Betel nut chewing (reference: Never)				
Former	− 1.650	0.586	− 0.137	0.975
Current	− 1.430	0.695	− 0.009	0.084
BMI (reference: Normal)				
Underweight	− 0.352	0.884	− 8.510	0.119
Overweight	− 1.310	0.340	1.130	0.446
Obesity	1.160	0.428	3.870	0.029
Secondhand smoke exposure (reference: No)				
Yes	− 1.990	0.241	3.790	0.152
Outdoor exercise*PM_2.5_		*p* value = 0.036		

*DLEC1* Deleted in lung and esophageal cancer 1, *PM* Particulate matter with diameter less than 2.5 microns, *SO*_*2*_ Sulfur dioxide, *CO* Carbon monoxide, *O*_*3*_ Ozone, *NOx* Nitrogen oxides, *BMI* Body mass index, *PM*_2.5_*Exercise interaction between PM_2.5_ and exercise

When we stratified PM_2.5_ concentrations into quartiles (Table 4), DLEC1 methylation in participants who exercised regularly was significant only at PM_2.5_ < 27.37 μg/m^3^ (*β* = − 5.280 × 10^−3^; *p* value = 0.009). The methylation was also significant at PM_2.5_ levels 31.82 ≤ PM_2.5_ < 38.73 μg/m^3^ (*β* = 3.980 × 10^−3^, *p* value = 0.009) for SO_2_, 27.37 ≤ PM_2.5_ < 31.82, and PM_2.5_ ≥ 38.73 μg/m^3^ (*β* = 3.850 × 10^−3^; *p* value = 0.003, *β* = − 1.700 × 10^−3^; *p* value = 0.035, respectively) for O_3_, and 31.82 ≤ PM_2.5_ < 38.73 and PM_2.5_ ≥ 38.73 μg/m^3^ (*β* = − 0.310 × 10^−3^; *p* value < 0.001 and *β* = − 0.320 × 10^−3^; *p* value = 0.003, respectively) for age.

**Table 4:** Multiple linear regression showing the association between outdoor exercise and DLEC1 promoter methylation stratified by PM_2.5_ quartiles

Variables	PM_2.5_ < 27.37		27.37 ≤ PM_2.5_ < 31.82		31.82 ≤ PM_2.5_ < 38.73		PM_2.5_ ≥ 38.73	
	*β*(× 10^−3^)	*p* value	*β*(× 10^−3^)	*p* value	*β*(× 10^−3^)	*p* value	*β*(× 10^−3^)	*p* value
Regular outdoor exercise (reference: No)								
Yes	− 5.280	0.009	0.323	0.872	− 1.280	0.433	1.680	0.428
SO_2_	5.900	0.275	− 0.940	0.121	3.980	0.009	0.434	0.622
CO	109.490	0.123	5.060	0.784	− 19.800	0.291	− 21.790	0.244
O_3_	0.180	0.724	3.850	0.003	0.725	0.059	− 1.700	0.035
NO_X_	− 1.640	0.105	0.744	0.201	0.252	0.323	− 0.557	0.376
Sex (reference: Women)								
Men	1.580	0.427	− 1.150	0.563	3.960	0.037	0.711	0.743
Age	− 0.095	0.286	− 0.150	0.098	− 0.310	< 0.001	− 0.320	0.003
Cigarette smoking (reference: Never)								
Former	0.510	0.861	3.440	0.213	− 0.542	0.818	5.950	0.063
Current	3.960	0.172	− 1.010	0.745	1.810	0.557	1.670	0.672
Alcohol drinking (reference: Never)								
Former	− 4.110	0.358	− 2.550	0.606	− 6.610	0.164	− 5.120	0.618
Current	4.380	0.183	0.484	0.900	6.970	0.056	− 6.840	0.093
Betel nut chewing (reference: Never)								
Former	− 10.91	0.135	1.990	0.672	− 2.710	0.485	− 7.910	0.276
Current	2.360	0.677	− 9.510	0.103	− 2.410	0.679	− 9.910	0.210
BMI (reference: Normal)								
Underweight	− 3.110	0.434	− 1.760	0.656	− 2.110	0.642	11.390	0.055
Overweight	− 2.750	0.206	− 1.650	0.457	− 0.709	0.699	2.010	0.345
Obesity	0.747	0.755	0.964	0.685	1.010	0.642	4.360	0.071
Secondhand smoke exposure (reference: No)								
Yes	1.590	0.548	− 1.520	0.573	− 3.030	0.379	1.010	0.761

*DLEC1* Deleted in lung and esophageal cancer 1, *PM* Particulate matter with diameter less than 2.5 microns, *SO*_*2*_ Sulfur dioxide, *CO* Carbon monoxide, *O*_*3*_ Ozone, *NOx* Nitrogen oxides, *BMI* Body mass index

Further stratification by exercise habits and PM_2.5_ quartiles revealed significant lower DLEC1 promoter methylation levels in one stratum (regular exercise at PM_2.5_ levels < 27.37 μg/m^3^): *β* = − 5.160 × 10^−3^; *p* value = 0.007 (Table 5).

**Table 5:** Multiple linear regression showing DLEC1 promoter methylation in relation to regular exercise habits and PM_2.5_ quartiles

Variables	*β*(× 10^−3^)	*p* value
Regular outdoor exercise, PM_2.5_ (reference: No regular outdoor exercise, PM_2.5_ ≥ 38.73)		
No regular outdoor exercise, 31.82 ≤ PM_2.5_ < 38.73	2.150	0.198
No regular outdoor exercise, 27.37 ≤ PM_2.5_ < 31.82	0.136	0.934
No regular outdoor exercise, PM_2.5_ < 27.37	− 0.808	0.634
Regular outdoor exercise, PM_2.5_ ≥ 38.73	1.310	0.444
Regular outdoor exercise, 31.82 ≤ PM_2.5_ < 38.73	0.570	0.750
Regular outdoor exercise, 27.37 ≤ PM_2.5_ < 31.82	0.410	0.827
Regular outdoor exercise, PM_2.5_ < 27.37	− 5.160	0.007
SO_2_	0.209	0.544
CO	− 6.270	0.379
O_3_	− 0.010	0.964
NO_X_	0.038	0.759
Sex (reference: Women)		
Men	0.652	0.490
Age	− 0.207	< 0.001
Cigarette smoking (reference: Never)		
Former	1.470	0.274
Current	2.340	0.123
Alcohol drinking (reference: Never)		
Former	− 3.390	0.174
Current	1.690	0.336
Betel nut chewing (reference: Never)		
Former	− 1.820	0.459
Current	− 4.270	0.145
BMI (reference: Normal)		
Underweight	− 1.070	0.620
Overweight	− 0.264	0.791
Obesity	1.620	0.143
Secondhand smoke exposure (reference: No)		
Yes	− 0.309	0.826

*DLEC1* Deleted in lung and esophageal cancer 1, *PM* Particulate matter with diameter less than 2.5 microns, *SO*_*2*_ Sulfur dioxide, *CO* Carbon monoxide, *O*_*3*_ Ozone, *NOx* Nitrogen oxides, *BMI* Body mass index

## Discussion

Based on the available literature, there are gaps in research focusing on the relationship between DLEC1 methylation and PM_2.5_ exposure. To our utmost knowledge, the current study is the first to investigate such a relationship. PM_2.5_ exposure was significantly associated with DLEC1 hypermethylation. Exercise seemingly modulated this relationship. That is, in relation to exercise, DLEC1 methylation was significantly associated with PM_2.5_ at levels < 27.37 μg/m^3^. In other words, the relationship between DLEC1 methylation and regular exercise disappeared as PM_2.5_ levels increased, implying that exercise might significantly influence DLEC1 methylation only at lower levels of PM_2.5._ This suggests that taking exercise when PM_2.5_ levels are high might expose people to more PM_2.5_ pollution, thereby abating the benefits of regular exercise. That is, the benefits of regular exercise might disappear when PM_2.5_ concentrations increase. Thus, people should not be encouraged to exercise when PM_2.5_ levels are high.

PM_2.5_ induces the generation of ROS [1], which when unbalanced, could have oncogenic consequences [1], like immune response stress, inflammatory injury, DNA damage, and oxidative stress [1–3]. ROS imbalance results from improperly regulated ROS production [2]. It also occurs when free radicals do not properly neutralize or detoxify oxidative effects [2]. Oxidative stress resulting from ROS is believed to be the main driving force for most air pollution-related adverse health effects [40]. PM_2.5_ is a critical issue that has increased oral, lung, breast, ovarian, and hepatic cancer morbidity and mortality in Taiwan [4, 50–52].

DLEC1 exhibits its cancer-inhibiting potentials by decreasing the invasiveness and metastasis of tumor cells [21, 22, 53] and also by enhancing apoptosis and arresting the G1 phase of the cell cycle [54, 55]. However, an abnormal methylation profile (like DLEC1 hypermethylation) is significantly linked to head and neck, ovarian, lung, renal, nasopharyngeal, oral, adrenocortical, hepatocellular, esophageal, gastric, and squamous cell carcinoma [11, 28, 30–32, 38, 39, 53, 54, 56–64]. In light of this, we believe that the PM_2.5_-related DLCE1 hypermethylation observed in our study could also heighten the risk of cancer.

Transcriptional suppression of DLEC1 by promoter hypermethylation is also believed to be an early event in carcinogenesis [62]. This is evident in adrenocortical, lung, nasopharyngeal, and esophageal squamous cell carcinoma [26, 30, 31, 57, 61]. Upregulation of DLEC1 is associated with reduced growth and invasiveness while downregulation is associated with cell proliferation, invasiveness, and poor disease prognosis [22, 31, 53, 58, 62]. DLEC1 expression could be restored with demethylating agents when downregulated, as demonstrated in lung, prostate, oral, nasopharyngeal, renal, ovarian, adrenocortical, and uterine tumors [11, 22, 25, 31, 53, 60–62, 65]. This reversible nature of DLEC1 suggests that it could be a potential treatment target for these cancers.

The detrimental effects of air pollution on health could be attenuated by exercise [18]. This could be achieved, in part, through protective immunologic responses and DNA methylation [18]. In our study, DLEC1 methylation was inversely associated with exercise, suggesting that exercise could attenuate PM_2.5_-related DLEC1 hypermethylation and the subsequent adverse effects. The possible mechanism through which exercise could modulate PM_2.5_-induced DLEC1 methylation is unclear. As previously stated, PM_2.5_ exacerbates inflammation and oxidative stress that could induce epigenetic alterations, especially DNA methylation [3, 66, 67]. Conversely, exercise regulates inflammation [6–9] and protects against air pollution-related health outcomes through altered DNA methylation profiles [18]. Inflammation is suggested as a possible contributor to epigenetic changes resulting from exercise interventions [66]. For instance, available literature corroborates the idea that exercise-associated inflammatory effects could be among the mechanisms that regulate DNA methylation [68]. Therefore, exercise might affect DLEC1 methylation by suppressing PM_2.5_-induced inflammation.

Previously, smoking and DLCE1 methylation were not significantly related [11, 28, 34]. It is important to note that in our study, smoking, SO_2_, CO, O_3_ showed significant associations with DLEC1 only after PM_2.5_ was stratified into quartiles. The attainment of a significant association between DLEC1 methylation and smoking after stratification into quartiles implies that PM_2.5_ might aggravate smoking-related DLEC1 methylation effects. Therefore, smoking which is already a harmful lifestyle habit could be more detrimental in air polluted areas. So far, the relation of age with DLEC1 methylation has not been concordant. For example, in esophageal cancer, age was significantly associated with DLCE1 methylation and expression [57]. On the other hand, in lung and gastric cancer, both factors had no significant association [11, 29]. In the current study, we found significant inverse associations between DLEC1 methylation and age, suggesting that DLEC1 methylation might decrease with increasing age. Age was also inversely associated with DNA methylation in a genome-wide DNA methylation study [69]. In the context of the current study, the underpinning mechanism cannot be clearly stated.

DNA methylation is a reliable molecular predictor of cancer because it is the most common epigenetic variation that could be detected even at premalignant or early malignant stages [13, 70]. Moreover, in noncancerous tissues, it could reveal previous exposure to carcinogens and so, is a possible indicator of disease risk [71]. Hence, the observed DLEC1 hypermethylation due to PM_2.5_ and smoking might serve as an early predictor of adverse health conditions. Moreover, hypomethylation of DLEC1 due to regular exercise at low PM_2.5_ levels shows that exercise intervention could help reverse the methylation status of DLEC1 and possibly upregulate the gene. As a limitation, we could not adjust for occupational exposure because we did not have related information. Moreover, we did not evaluate DLEC1 expression due to the unavailability of data in the TWB dataset. Nevertheless, many studies found significant associations between DLEC1 hypermethylation and downregulation [26, 30, 31, 57, 61].

## Conclusions

This study shows that PM_2.5_ might affect DLEC1 methylation in individuals with no personal history of cancer. Exercise might regulate the effect of PM_2.5_ on DLEC1 methylation, especially at low concentrations of PM_2.5_. Our findings support prior reports that DNA methylation in noncancerous tissues could reveal previous exposure to carcinogens. Hence, DLEC1 promoter hypermethylation might elucidate the epigenetic mechanism through which PM_2.5_ enhances disease onset and might be a possible biomarker of disease risk.

## Data Availability

The data that support the findings of this study are available from Taiwan Biobank but restrictions apply to the availability of these data, which were used under license for the current study, and so are not publicly available. Data are however available from the authors upon reasonable request and with permission of Taiwan Biobank.

## Abbreviations

DLEC1: Deleted in lung and esophageal cancer 1
PM: Particulate matter
PM_2.5_: Particulate matter (PM) < 2.5 μm
SO_2_: Sulfur dioxide
CO: Carbon monoxide
O_3_: Ozone
NOx: Nitrogen oxides
*β*: Beta coefficient
DNA: Deoxyribonucleic acid
ROS: Reactive oxygen species
CpG: Cytosine-phosphate-guanine
SE: Standard error
BMI: Body mass index
AQMD: Air Quality Monitoring Database
TWB: Taiwan Biobank
